# Supplementary material for: Splice-Junction-Based Mapping of Alternative Isoforms in the Human Proteome
Source: Cell Rep. Author manuscript; Available in PMC 2020 Jan 15. (PMC6961840; doi:10.1016/j.celrep.2019.11.026)
Supplement: 2 [file NIHMS1546469-supplement-2.zip › DF1/PXD010154/Lung/Lung_38_NASP_AAVEGTVEAGATVESTALK.pdf]

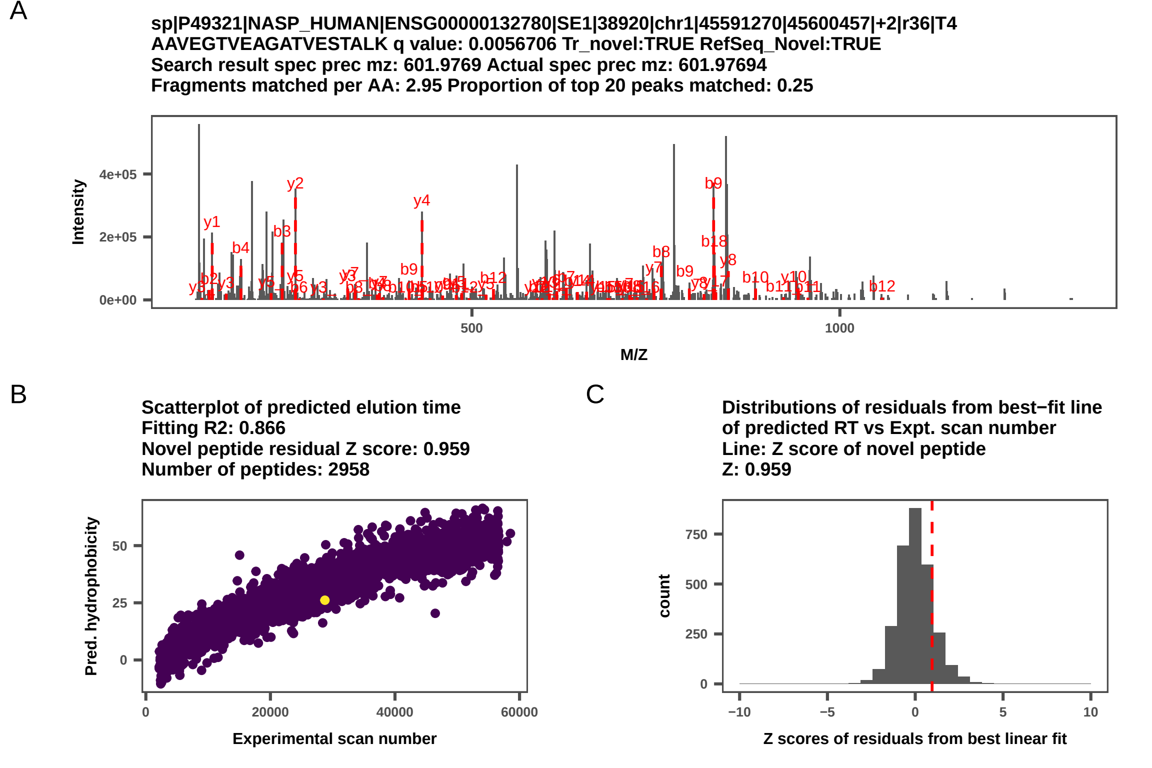
A
sp|P49321|NASP_HUMAN|ENSG00000132780|SE1|38920|chr1|45591270|45600457|+2|r36|T4
AAVEGTVEAGATVESTALK q value: 0.0056706 Tr_novel:TRUE RefSeq_Novel:TRUE
Search result spec prec mz: 601.9769 Actual spec prec mz: 601.97694
Fragments matched per AA: 2.95 Proportion of top 20 peaks matched: 0.25
Intensity
4e+05
2e+05
0e+00
500
1000
M/Z
y1
y2
b3
b4
y4
b9
b18
b8
y7
y8
b10
y10
b12
B
Scatterplot of predicted elution time
Fitting R2: 0.866
Novel peptide residual Z score: 0.959
Number of peptides: 2958
Pred. hydrophobicity
50
25
0
0
20000
40000
60000
Experimental scan number
C
Distributions of residuals from best−fit line
of predicted RT vs Expt. scan number
Line: Z score of novel peptide
Z: 0.959
count
750
500
250
0
−10
−5
0
5
10
Z scores of residuals from best linear fit
